# Clinical Features and Resistance to Entecavir Monotherapy of Patients with Hepatitis B

**DOI:** 10.1155/2021/3259833

**Published:** 2021-08-10

**Authors:** Hideo Takayama, Takuya Komura, Takashi Kagaya, Saiho Sugimoto, Noriaki Orita, Yoshiro Asahina, Masashi Nishikawa, Hajime Ohta, Shuichi Kaneko, Masashi Unoura

**Affiliations:** ^1^Division of Gastroenterology NHO, Kanazawa Medical Center, 1-1 Shimoishibikimachi, Kanazawa, Ishikawa 920-8650, Japan; ^2^System Biology, Kanazawa University, Graduate School of Medical Science, Kanazawa, Japan; ^3^Municipal Tsuruga Hospital, Tsuruga, Japan

## Abstract

**Aim:**

Hepatitis B virus (HBV) infection is a major public health concern worldwide. Entecavir (ETV), a first-line nucleos(t)ide analogue (NA) for HBV, has a low risk of resistance. We evaluated the efficacy of ETV monotherapy, ratio of ETV-resistant, and the clinical features of patients with ETV resistance.

**Methods:**

A total of 130 patients (72 males, 58 females; mean age, 61 ± 15 years) were divided into a NA-naïve group (*n* = 108) and NA-experienced group (*n* = 22). We examined the clinical outcomes of ETV monotherapy and associated factors. We also assessed the clinical features of 15 patients with resistance to ETV (mean, 51.0 ± 27.4 weeks).

**Results:**

Among the 130 patients, 94.1% achieved ALT normalization and 63.6% achieved serum HBV DNA negativity after ETV monotherapy for 96 weeks. Of the patients in the NA-naïve group, 93.1% and 60.4% achieved ALT normalization and HBV DNA negativity, respectively. Of the patients in the NA-experienced group, 100% and 74.9% achieved ALT normalization and HBV DNA negativity, respectively. Compared to patients on ETV continuously, 15 ETV-resistant patients had a higher baseline HBV viral load. There was a significant difference in the time to HBV DNA negativity, but not ALT normalization after ETV monotherapy in these groups. Rescue treatment with other NAs led to ALT normalization in all of these patients, but not HBV DNA negativity.

**Conclusions:**

ETV monotherapy has a long-term clinical efficacy. While some patients especially with HBV DNA high viral load developed ETV resistance, rescue treatment led to ALT normalization in these patients.

## 1. Introduction

Hepatitis B virus (HBV) infection is a leading cause of chronic hepatitis, liver cirrhosis, and hepatocellular carcinoma (HCC), all of which ultimately result in death [1]. Over 300 million people are chronically infected with HBV worldwide [2]. Treatment of HBV leads to prevention of complications of chronic liver diseases such as HCC [3]. It has also been reported that there is a potential effect of alcohol intake on the progression of liver disease in patients with HBV hepatitis [4]. The American Association for the Study of the Liver (AASLD) [5], European Association for the Study of the Liver (EASL) [6], Asian Pacific Association for the Study of the Liver (APASL) [7], and Japan Society of Hepatology (JSH) [8] have published guidelines for diagnosing, preventing, and managing HBV infection. The majority of patients with persistent HBV infection may not require antiviral therapy, the indications for which are based on age, histological progression, the alanine aminotransferase (ALT) level, and the HBV DNA level [5–8]. Therefore, therapy should be considered in patients with more active or advanced liver disease and in those likely to respond in accordance with defined treatment endpoints. Treatment algorithms have been developed to assist in the identification of suitable candidates for treatment and to determine when to initiate treatment [5–8].

Six antiviral nucleos(t)ide analogues (NAs)—lamivudine (LAM), adefovir dipivoxil (ADV), entecavir (ETV), telbivudine (LdT), tenofovir disoproxil fumarate (TDF) and tenofovir alafenamide fumarate (TAF)—have been approved for chronic HBV hepatitis. All six are HBV polymerase/reverse transcriptase (RT) inhibitors and suppress HBV replication [9, 10]. Because ETV, like TDF and TAF, decreases the HBV viral load, promotes ALT normalization, and has a low risk of viral resistance, it has been used as a first-line therapy [5, 11, 12]. ETV (0.5 mg per day) has been approved for treatment-naïve cases in Japan since 2006 [8]. A major concern with long-term NA treatment is antiviral resistance mutations [13]. Because drug-resistant mutant HBV populations expand via replication, antiviral therapy should aim to suppress viral replication as completely and rapidly as possible [9, 10]. However, the clinical features of patients with ETV resistance are unclear.

We retrospectively examined the outcomes of patients with HBV hepatitis who received ETV monotherapy and the clinical features and rescue treatment outcomes of patients with ETV resistance.

## 2. Methods

This study was performed according to the ethical guidelines of the Declaration of Helsinki (1964, and its later amendments); the protocol was approved by the Institutional Review Board of NHO Kanazawa Medical Center (Kanazawa, Japan).

### 2.1. Patients

A total of 316 patients with HBV hepatitis received NA therapy at NHO Kanazawa Medical Center from December 2000 to December 2019. Of them, 130 patients with HBV hepatitis (72 males, 58 females; mean age, 60.9 ± 15.0 years; mean follow-up duration, 69.4 (0–163) months) who received ETV monotherapy were enrolled in this study after providing informed consent. We have confirmed that there is no coinfection with HCV in this study.

We applied NA therapy for patients with HBV hepatitis according to the JSH guidelines for the management of HBV infection [8]. The treatment indications are based on the serum ALT level, HBV DNA level, and extent of liver fibrosis.

We defined ETV resistance as virological breakthrough (>1 log_10_ increase in the serum HBV DNA level from nadir after an initial virological response) or insufficient viral suppression [5]. ETV monotherapy was applied for NA-naïve and NA-experienced patients.

### 2.2. Clinical Features

At admission, we evaluated the following factors: age, gender, baseline aspartate aminotransferase level (AST), highest ALT level, prior NA experience, and HBsAg positivity. Data on the baseline HBV DNA and HBcrAg levels, viral genotype, extent of liver fibrosis, and response to ETV monotherapy are given in Table 1.

### 2.3. Genotypic Resistance Assay

The PCR-invader assay (BML Corp., Tokyo, Japan) was used to detect resistance mutations [14].

### 2.4. Statistical Analysis

Statistical analysis was performed using Prism software (GraphPad Software Inc., San Diego, CA, USA). Data are provided as medians and interquartile ranges or as means and standard errors of the mean. Between-group differences were assessed by the Mann–Whitney *U* test or the *χ*^2^ test. The probability of ALT normalization and HBV DNA negativity were examined by the Kaplan–Meier method, and differences were assessed by the log-rank test. Factors associated with ALT normalization and HBV DNA negativity were subjected to univariate and multivariate analyses using the Cox proportional hazards model.

## 3. Results

### 3.1. Outcomes of ETV Monotherapy

**Figure 1:**
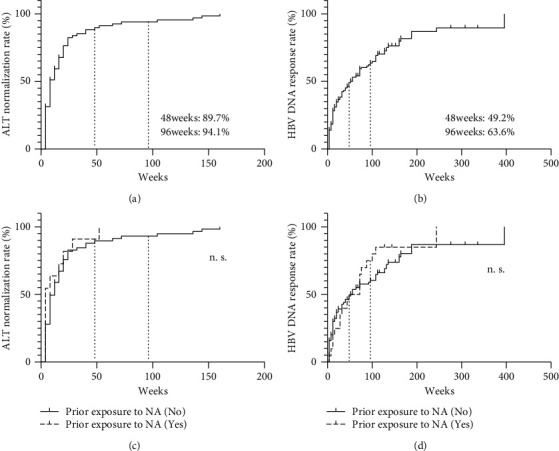
Outcome of ETV monotherapy. Of the 130 patients, ETV monotherapy led to ALT normalization in 89.7% and 94.1% after 48 and 96 weeks, respectively (a). ETV monotherapy led to serum HBV DNA negativity in 49.2% and 63.6% of the patients at 48 and 96 weeks, respectively (b). There was no significant difference between the NA-naïve group (*n* = 108) and NA-experienced group (*n* = 22) in the rate of ALT normalization (c) or serum HBV DNA negativity (d).

First, we examined the probability of and time to normalization of the ALT level and HBV DNA negativity after 48 or 96 weeks of ETV monotherapy. Among the 130 patients, 89.7% and 94.1% achieved ALT normalization after ETV monotherapy for 48 and 96 weeks, respectively (Figure 1(a)). Moreover, 49.2% and 63.6% of the patients achieved serum HBV DNA negativity after ETV monotherapy for 48 and 96 weeks, respectively (Figure 1(b)). Next, we divided the patients into an NA-naïve group (*n* = 108) and NA-experienced group (*n* = 22). Of the patients in the NA-naïve group, 89.6% and 93.1%, and 48.8% and 60.4% achieved ALT normalization and serum HBV DNA negativity after ETV monotherapy for 48 and 96 weeks, respectively. Of the patients in the NA-experienced group, 90.9% and 100%, and 49.9% and 74.9%, achieved ALT normalization and serum HBV DNA negativity after ETV monotherapy for 48 and 96 weeks, respectively. There was no significant difference between the NA-naïve and NA-experienced groups in the rate of ALT normalization (Figure 1(c)) or serum HBV DNA negativity (Figure 1(d)).

### 3.2. Features of Patients with ETV Resistance

**Figure 2:**
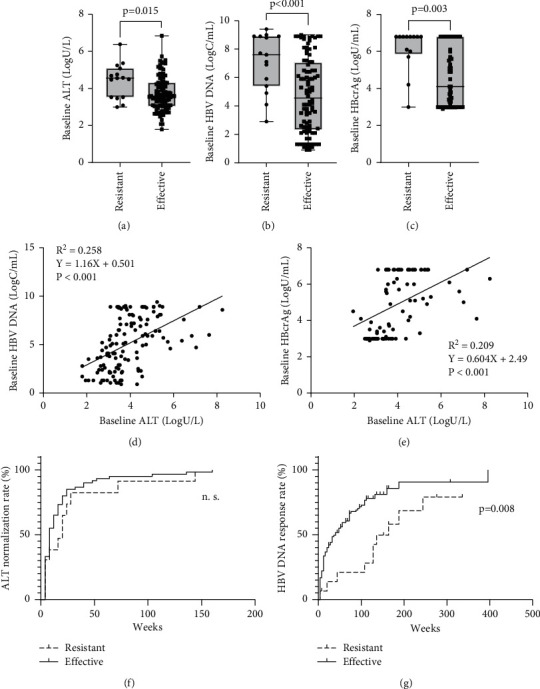
Correlation between time to ETV monotherapy resistance and pretreatment clinical features. In the ETV-resistant group, the proportion of young patients and the ALT (a), HBV DNA (b), and HBcrAg (c) levels at baseline were significantly higher than those in the ETV-responsive group. Baseline ALT levels were significantly correlated with the baseline HBV DNA (d) and HBcrAg (e) levels, irrespective of ETV resistance. There was no significant group difference in the time to ALT normalization (f), but there was a significant group difference in the time to HBV DNA negativity (*p*=0.008) (g).

Of the 130 patients, 112 (86.2%) were followed for more than 24 weeks. Of the 112 patients, 15 (13.4%) had resistance to ETV and 97 (86.6%) did not. The characteristics of the patients are listed in Table 2. The ETV-resistant group were younger and more likely to be male and HBeAg positive and had significantly higher baseline ALT (Figure 2(a)), HBV DNA (Figure 2(b)), and HBcrAg (Figure 2(c)) levels. However, there was no significant difference between the two groups in prior NA experience, proportion of genotype C patients, or extent of liver fibrosis. In addition, there was a significant correlation between the serum HBV DNA and ALT levels (Figure 2(d)) and between the serum HBcrAg and ALT levels (Figure 2(e)). There was no significant difference in the time to ALT normalization (Figure 2(f)), but there was a significant difference in the time to HBV DNA negativity after ETV monotherapy in these groups (*p*=0.008) (Figure 2(g)).

The multivariate analysis of the effect of ETV resistance included the risk factors for ETV resistance in univariate analyses (Table 3). Baseline ALT (≥3 × ULN, *p*=0.004) and HBcrAg level (≥5 log U/mL, *p*=0.013) were significantly associated with ETV resistance (Table 3).

### 3.3. Time to ETV Resistance

Next, we examined the clinical features of the 15 patients with ETV resistance (12 men and 3 women; mean age, 49.1 ± 12.7 years) (Tables 4 and 5). Of these patients, 3 and 12 experienced with and naïve to NA therapy, respectively. Although genotypic resistance mutation was not detected in the 15 patients before ETV monotherapy, genotypic resistance (L180M + S202G + M204V) was detected in an NA-naïve patient after NA monotherapy failure.

For the 15 patients with ETV resistance, we added an NA or switched to a different one. For five patients, we added TAF (one patients) or TDF (four patients), while nine patients were switched to TAF (eight patients) or TDF (one patient) monotherapy. Also, one patient was switched to LAM + ADV therapy. The dosage for rescue treatment was the dose listed in the package insert for each drug: 25 mg once daily for TAF, 300 mg once daily for TDF, 100 mg once daily for LAM, and 10 mg once daily for ADV. However, for patients with impaired renal function, the dose was reduced to the specified level. The median duration of rescue treatment was 36 months (IQR: 25.5–46.5), and HBV DNA and ALT levels were assessed at least once every 3 months.

**Figure 3:**
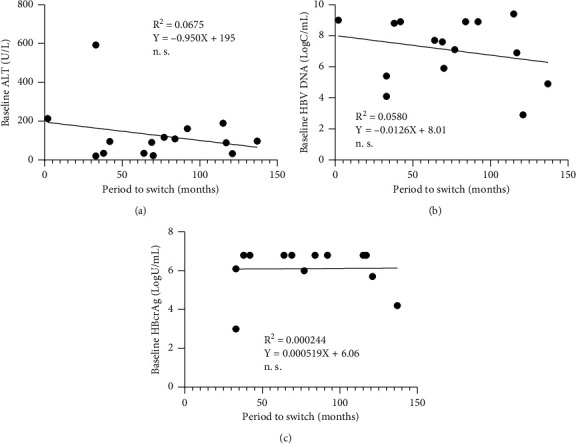
Factors associated with the time taken to switch to NA therapy from ETV monotherapy. The baseline ALT (a), HBV DNA (b), and HBcrAg (c) levels were not significantly correlated with the time taken to switch to NA therapy.

We analyzed factors associated with the time taken to switch to NA therapy from ETV monotherapy. The baseline ALT (Figure 3(a)), HBV DNA (Figure 3(b)) and HBcrAg (Figure 3(c) levels were not correlated with the time taken to switch to NA therapy. Although ALT elevation was observed in four patients, switching to NA therapy led to its normalization (mean, 51.0 ± 27.4 weeks). HBV DNA elevation (>3.0 log U/mL) was also observed in four patients, but all of them achieved an HBV DNA level of <3.0 log U/mL (mean, 16.0 ± 6.3 weeks).

### 3.4. Differences between the NA-Naïve and NA-Experienced Groups in Patients with ETV Resistance

There were significant differences between the NA-naïve and NA-experienced groups in pretreatment HBV DNA (*p*=0.011) and HBcrAg (*p*=0.021) levels (Table 6). The NA-experienced HBV patients showed a tendency toward ETV resistance even with a low HBV viral load.

## 4. Discussion

Although most patients with HBV infection who receive ETV monotherapy have a benign clinical course, some develop ETV resistance. A major concern with long-term NA treatment is antiviral resistance mutations. ETV, the first approved oral NA, develops resistance at a very low rate in treatment-naïve patients, although the rate of ETV resistance increases to 51% in patients with resistance to LAM [9]. Thus, detecting resistant variants is critical for appropriate patient treatment, and some substitutions have been characterized in an ETV-refractory patient, which mediated ETV resistance by significantly reducing viral replication [15]. However, evaluating all known resistance mutations is difﬁcult, expensive, and time-consuming [16]. In fact, we detected genetic resistance mutation in only one patient in this study, although the PCR-Invader assay reported that 50.6% of the samples were positive for ETV resistance mutations [14]. Therefore, physicians should monitor the HBV viral load even without examining resistance variants because the rate of viral suppression increases over time and the time required for treatment adaptation depends on viral load decay, especially in patients starting from a very high viral load who may need therapy for a few more weeks to achieve an undetectable HBV DNA level. A persistent low viremia level and plateau thereof indicate a need for treatment optimization to maximize viral suppression and minimize the subsequent risk of resistance [17]. The AASLD guidelines state that the rate at which resistant variants are selected is related to the pretreatment serum HBV DNA level, rapidity of viral suppression, duration of treatment, prior exposure to NA therapies, and most importantly, the genetic barrier to resistance of the NA [5]. Our results also suggested that patients with a high pretreatment HBV DNA viral load tend to develop resistance to ETV.

ETV is associated with resistance in LAM-experienced (particularly LAM-refractory) patients, and bone and renal safety issues are a concern with TDF [18, 19]. Therefore, it has been proposed that optimizing the use of TAF is suitable for NA therapy along with guidance on specific patient groups at risk of renal or bone disease LAM-experienced patients [20, 21]. As for ETV, it has a large number of accumulating evidences, usage experiences, and cost effectiveness. It will likely continue to be the first-line NAs for HBV infection, except for LAM-experienced patients and those with a high pretreatment HBV viral load.

In this study, 15 patients had to switch to other NAs because of resistance to ETV. The choice of rescue NA therapy has been widened due to the availability of new NAs. Tenofovir (TDF or TAF) is added to ETV monotherapy in the presence of ETV resistance, as the preferred strategy to control viral replication in the majority of patients in whom liver disease progression is attenuated [22]. In particular, TAF monotherapy is used as rescue therapy to normalize ALT; TAF is preferred because it has fewer adverse effects (such as renal and bone damage) than TDF.

This study had several limitations. First, the indications for ETV monotherapy for HBV infection have changed due to the development of TDF and TAF, but ETV monotherapy is even now the first-line regimen for HBV infection in Japan. Second, this was a retrospective medical record-based study performed at a single center, the sample size was small, and the detailed drinking history was not reviewed. In addition, it may be possible that there is a problem with drug adherence because the percentage of ETV resistance emerged in this study was higher than previously reported.

In conclusion, patients with HBV hepatitis who received ETV monotherapy had satisfactory outcomes. If the pretreatment HBV DNA viral load is high, the serum HBV DNA and ALT levels should be more carefully monitored, and TAF therapy shall be taken in account in the future.

## Figures and Tables

**Table 1:** Baseline clinical characteristics of ETV monotherapy hepatitis B patients.

Characteristics	ETV monotherapy (*n* = 130)
Age at the beginning (years, (median, IQR))	62.0 (51.0–71.3)
Gender (M/F (*n*, %))	72 (55.4)/58 (44.6)
Baseline AST (U/L (median, IQR))	37.0 (23.8–75.5)
Baseline ALT (U/L (median, IQR))	37.5 (22.0–95.5)
Prior NA exposure (yes/no (*n*, %))	22 (16.9)/108 (83.1)
Baseline HBeAg (+/−/unknown (*n*, %))	51 (39.2)/65 (50.0)/14 (10.8)
Baseline HBV DNA (LogC/mL (median, IQR))	5.1 (2.5–7.7)
Baseline HBcrAg (LogU/mL (median, IQR))	5.1 (3.0–6.8)
Genotype (*A*/*B*/*C*/*D*/unknown (*n*, %))	4 (3.1)/14 (10.8)/70 (53.8)/1 (0.8)/41 (31.5)
Fibrosis stage (*F*1/*F*2/*F*3/*F*4/unknown (*n*, %))	7 (5.4)/27 (20.8)/12 (9.2)/3 (2.3)/81 (62.3)
Response to the therapy (resistant/effective/excluded (*n*, %))	15 (11.5)/97 (74.6)/18 (13.8)

**Table 2:** Comparison of clinical and histological characteristics between ETV monotherapy resistant and effective hepatitis B patients.

Characteristics	Resistant group (*n* = 15)	Effective group (*n* = 97)	*P* value
Age at the beginning (years (median, IQR))	48.0 (37.0–61.0)	63.0 (54.5–71.0)	*P* < 0.001
Gender (M/F (*n*, %))	12 (80.0)/3 (20.0)	48 (49.5)/49 (50.5)	*P*=0.027
Baseline AST (U/L (median, IQR))	65.0 (25.0–83.0)	35.0 (23.0–64.5)	n. s.
Baseline ALT (U/L (median, IQR))	95.0 (34.0–161.0)	36.0 (21.0–74.8)	*P*=0.015
Prior NA exposure (yes/no (*n*, %))	3 (20.0)/12 (80.0)	18 (18.6)/79 (81.4)	n. s.
Baseline HBeAg (+/−/unknown (*n*, %))	10 (66.7)/5 (33.3)/0 (0.0)	31 (32.0)/52 (53.6)/14 (14.4)	*P*=0.034
Baseline HBV DNA (LogC/mL (median, IQR))	7.6 (5.4–8.9)	4.4 (2.3–7.0)	*P* < 0.001
Baseline HBcrAg (LogU/mL (median, IQR))	6.8 (5.9–6.8)	4.1 (3.0–6.8)	*P*=0.003
Genotype (*A*/*B*/*C*/*D*/unknown (*n*, %))	0 (0.0)/3 (20.0)/12 (80.0)/0 (0.0)/0 (0.0)	3 (3.5)/10 (9.6)/50 (50.4)/1 (0.9)/33(40.0)	n. s.
Fibrosis stage (*F*1/*F*2/*F*3/*F*4/unknown (*n*, %))	3 (20.0)/7 (46.7)/1 (6.7)/1 (6.7)/3 (20.0)	4 (4.1)/19 (19.6)/9 (9.3)/3 (3.1)/62 (63.9)	n. s.

**Table 3:** Factors contributing to ETV monotherapy resistance (multivariate analysis).

Characteristics	Univariate OR (95% CI)	*P* value	Multivariate adjusted OR (95% CI)	*P* value
Age at the beginning (＜65 years)	4.8 (1.1–21.9)	*P*=0.044	—	—
Gender (male)	4.1 (1.2–14.1)	*P*=0.049	—	—
Baseline AST (≧2 × ULN)	3.0 (0.9–8.3)	*P*=0.070	—	—
Baseline ALT (≧3 × ULN)	5.4 (1.7–17.9)	*P*=0.004	3.3 (0.9–13.6)	*P*=0.083
Prior exposure to NA therapies (yes)	1.1 (0.3–4.3)	*P* > 0.999	—	—
Baseline HBeAg (positive)	3.4 (1.1–9.4)	*P*=0.047	—	—
Baseline HBV DNA (≧5 LogC/mL)	4.4 (1.3–15.3)	*P*=0.025	—	—
Baseline HBcrAg (≧5 LogU/mL)	7.3 (1.8–34.5)	*P*=0.013	4.7 (1.0–34.0)	*P*=0.070
Genotype C (yes)	1.1 (0.3–4.1)	*P* > 0.999	—	—
Fibrosis stage (≦*F*2)	5.2 (0.8–60.9)	*P*=0.141	—	—

**Table 4:** Clinical, epidemiologic, and histological characteristics of all ETV-resistant group patients.

No.	Age (years)	Gender	Prior exposure to NA	Biopsy	Genotype	Genotypic resistance (before ETV)	Genotypic resistance (after ETV)
1	52	Male	Yes/LAM	*F*3*A*2	C	Negative	Undetectable
2	40	Male	Yes/LAM	*F*1*A*1	C	Undetectable	Undetectable
3	30	Male	Yes/LAM + ADV	*F*2*A*1	C	—	Undetectable
4	44	Male	No	*F*2*A*2	B	—	—
5	76	Male	No	—	C	—	Undetectable
6	54	Male	No	—	B	—	Undetectable
7	66	Male	No	*F*1*A*2	C	—	L180M + S202G + M204V
8	36	Male	No	*F*2*A*2	C	Negative	Undetectable
9	61	Male	No	*F*4*A*2	B	Negative	Undetectable
10	35	Male	No	*F*1*A*1	C	—	Undetectable
11	37	Male	No	*F*2*A*2	C	Negative	Undetectable
12	61	Female	No	*F*2*A*2	C	—	Undetectable
13	48	Female	No	*F*2*A*1	C	Negative	Undetectable
14	55	Male	No	—	C	Negative	Undetectable
15	42	Female	No	*F*2*A*2	C	—	Undetectable

**Table 5:** Clinical, epidemiologic, and histological characteristics of all ETV-resistant group patients (2).

No.	Baseline			Period to switch (months)	Rescue antiviral treatment	At switch			ALT < 30 U/L after switch (weeks)	HBV DNA < 3 LogC/mL after switch (weeks)
	ALT (U/L)	HBV DNA (LogC/mL)	HBcrAg (LogU/mL)			ALT (U/L)	HBV DNA (LogC/mL)	HBcrAg (LogU/mL)		
1	32	2.9	5.7	121	ETV + TDF	42	5.7	—	97	12
2	20	4.1	3	33	LAM + ADV	19	2.6	—	—	—
3	592	5.4	6.1	33	TDF	18	2.1	—	—	—
4	116	7.1	6	77	ETV + TDF	19	4.2	3	—	8
5	213	9	—	2	ETV + TDF	1144	8.1	—	46	20
6	22	5.9	—	70	ETV + TDF	19	1.7	2.9	—	—
7	95	8.9	6.8	42	ETV + TAF	25	6.9	7	—	24
8	161	8.9	6.8	92	TAF	33	1.7	5.5	28	—
9	97	4.9	4.2	137	TAF	18	1.7	2.9	—	—
10	34	8.8	6.8	38	TAF	18	2.8	7	—	—
11	189	9.4	6.8	115	TAF	20	1.7	4.1	—	—
12	34	7.7	6.8	64	TAF	10	1.7	4.9	—	—
13	108	8.9	6.8	84	TAF	20	1.7	5.7	—	—
14	88	6.9	6.8	117	TAF	28	1.7	4	—	—
15	90	7.6	6.8	69	TAF	45	1.7	5.7	33	—

**Table 6:** Comparison of clinical characteristics of ETV-resistant hepatitis B patients between NA-naïve patients and patients with prior NA exposure.

Characteristics	NA naive patients (*n* = 12)	Patients with prior NA exposure (*n* = 3)	*P* value
Age at the beginning (years)	51.3 ± 12.6	40.7 ± 9.0	n. s.
Gender (M/F (*n*, %))	9 (75)/3 (25)	3 (100)/0 (0)	n. s.
Baseline AST (U/L)	64.1 ± 29.2	84.0 ± 87.0	n. s.
Baseline ALT (U/L)	103.9 ± 57.5	214.7 ± 266.9	n. s.
Baseline HBV DNA (LogC/mL)	7.8 ± 1.4	4.1 ± 1.0	*P*=0.011
Baseline HBeAg (+/− (*n*, %))	8 (66.6)/4 (33.3)	2 (66.6)/1 (33.3)	n. s.
Baseline HBcrAg (Log U/mL)	6.5 ± 0.8	4.9 ± 1.4	*P*=0.021
Genotype (*B*/*C* (*n*, %))	3 (25.0)/9 (75.0)	0 (0.0)/3 (100.0)	n. s.
HCC (*n*, %)	2 (16.7)	0 (0)	n. s.
Fibrosis stage (*F*1/*F*2/*F*3/*F*4/unknown (*n*, %))	2 (16.7)/6 (50.0)/0 (0.0)/1 (8.3)/3 (25.0)	1 (33.3)/1 (33.3)/1 (33.3)/0 (0.0)/0 (0.0)	n. s.
Period to next treatment (months)	75.6 ± 36.0	62.3 ± 41.5	n. s.
Genotypic resistance (after ETV) (detected/undetectable/no sample (*n*, %))	1 (8.3)/10 (83.3)/1 (8.3)	0 (0.0)/3 (100.0)/0 (0.0)	n. s.

## Data Availability

In order to protect personal information, the authors refrain from publishing patient-specific raw data beyond the data in table.
